# Diet–microbial cross–talk underlying increased visceral perception

**DOI:** 10.1080/19490976.2023.2166780

**Published:** 2023-01-19

**Authors:** Giada De Palma, David E. Reed, Premysl Bercik

**Affiliations:** a Farncombe Family Digestive Health Research Institute, McMaster University, Hamilton, Ontario, Canada; b GI Diseases Research Unit, Queens University, Kingston, Ontario, Canada

**Keywords:** diet, microbiome, abdominal pain, visceral hypersensitivity, irritable bowel syndrome, FODMAPs

## Abstract

Visceral hypersensitivity, a fundamental mechanism of chronic visceral pain disorders, can result from both central or peripheral factors, or their combination. As an important regulator of normal gut function, the gut microbiota has been implicated as a key peripheral factor in the pathophysiology of visceral hypersensitivity. Patients with chronic gastrointestinal disorders, such as irritable bowel syndrome, often present with abdominal pain secondary to adverse reactions to dietary components. As both long- and short-term diets are major determinants of gut microbiota configuration that can result in changes in microbial metabolic output, it is becoming increasingly recognized that diet–microbiota interactions play an important role in the genesis of visceral sensitivity. Changes in pain signaling may occur via diet-induced changes in secretion of mediators by both the microbiota and/or host cells. This review will examine the peripheral influence of diet–microbiota interactions underlying increased visceral sensitivity.

## Introduction

Visceral hypersensitivity, defined as exaggerated pain signaling in response to stimuli within visceral organs such as the gastrointestinal (GI) tract, is a fundamental mechanism of chronic visceral pain disorders.^1–4^ The gut microbiota contributes to regulation of normal gut function but has also been implicated in the pathophysiology of visceral hypersensitivity,^5–8^ although detailed mechanisms remain unclear. Diet is a major determinant of gut microbiota composition and function. Additionally, food intake induces both chemical (e.g. release of enteroendocrine mediators) and mechanical stimuli (e.g. bowel distension) within the GI tract capable of activating neural pathways. Yet, despite the multiple effects of diet, processing food within the gut is typically not consciously perceived. However, many patients with chronic GI disorders perceive food as a major trigger of their symptoms. For example, 25–84% of the patients with irritable bowel syndrome (IBS), a disorder characterized by abdominal pain and altered bowel habit in the absence of overt inflammation,^9^ cite food intolerances unleashing GI symptoms.^10–13^ While food-induced abdominal pain in GI disorders has traditionally been thought to be secondary to an already hypersensitive GI tract, recent studies suggest that dietary components can stimulate pathways that ultimately lead to visceral hypersensitivity. Since the effects of a particular diet differ from person to person, possibly influenced by a combination of host and microbiome features,^14^ the gut microbiota is considered an important intermediary between dietary factors and changes in visceral sensitivity. This review will examine the role of diet–microbiota interactions that lead to increased visceral sensitivity.

## Sensory signaling in the GI tract

Sensory information from the GI tract to the central nervous system (CNS) follows two main routes: (1) vagal afferent nerves, whose cell bodies lie within the nodose ganglia and (2) spinal afferents, whose cell bodies lie within the dorsal root ganglia (DRG). Vagal afferent nerves are more prevalent in the proximal gut, although studies suggest there is some innervation by vagal afferents in the colon.^15^ These nerves are primarily involved in the transmission of physiological stimuli, such as satiety signaling or ongoing motor activity within the gut.^16^ Spinal afferent nerves innervate the entire GI tract via both splanchnic and pelvic nerves.^17^ While subtypes of these afferent nerves may be involved in reflex pathways within the GI tract, other subtypes of spinal afferent nerves are involved in transmission of nociceptive signals to the CNS.^18,19^

Visceral hypersensitivity can occur via central or peripheral mechanisms. Central sensitization can occur at different levels within the CNS. This may include increased activation of neurons within the dorsal horn of the spinal cord,^20^ increased synaptic transmission of ascending pathways or decreased inhibition of descending pathways within the spinal cord,^21^ or changes in processing of sensory input within the brain.^22^ In this review, however, we will concentrate on the peripheral mechanisms of visceral pain, as the focus will be on diet and microbiota interactions.

Sensitivity of spinal afferent nerves innervating the GI tract is the result of a balance of activation of excitatory and inhibitory pathways (reviewed in Aguilera-Lizarraga *et al*.^23^). Visceral hypersensitivity can result when there is an imbalance toward excitatory pathways. This is the case when there is ongoing inflammation, for example, as many inflammatory and immune mediators, acting via G-protein coupled receptors that in turn modulate ion channels, increase excitability of spinal nerves. Consequently, a stimulus (e.g. colonic distension) will now evoke a greater response resulting in either the sensation of abdominal pain following a normally innocuous stimulus (i.e. allodynia) or greater abdominal pain following a painful stimulus (i.e. hyperalgesia). While sensitization of afferent nerves within the gut can be short-lived, there can be long-lasting sensitization following resolution of inflammation. This may be the case for visceral hypersensitivity in patients with post-infectious IBS (PI-IBS)^24^ or inflammatory bowel disease (IBD) patients who continue to experience chronic abdominal pain, despite achieving endoscopic remission.^25^ Although several studies have shown evidence of residual low-grade inflammation or immune activation,^26^ the underlying mechanisms are still to be fully elucidated.

There is evidence that patients with IBS have increased excitatory mediators, such as proteases, prostaglandins, histamine and serotonin (5-HT), both within the tissue as well as within the gut lumen, and the continued presence of these mediators may be responsible for ongoing visceral hypersensitivity. Activated mast cells release multiple bioactive mediators, including histamine, prostaglandins and tryptase, and have long been implicated in the pathogenesis of IBS, particularly in the generation of visceral hypersensitivity. Increased number of colonic mast cells, often found in proximity to sensory nerves in IBS patients,^27,28^ have been linked to pain severity. Mucosal mast cell mediators excite nociceptive visceral sensory nerves, and this effect can be inhibited by histamine 1 receptor (H1R) blockade and serine protease inactivation.^29^ The H1R antagonist ebastine also decreased abdominal pain scores and improved global symptom relief in a small clinical study of IBS patients.^30^ Additionally, the H1R antagonist and mast cell stabilizer ketotifen has also been shown to improve visceral hypersensitivity in patients with IBS.^31^ Indeed, histamine release by mast cells, triggered by local immune response to specific food components,^32^ has been shown to promote visceral hypersensitivity through H1R-mediated TRPV1 sensitiation.^32^ Other mediators, as well, have been shown to contribute to the development of visceral hypersensitivity.^33,34^ A recent study demonstrated that intracolonic infusion of IBS fecal supernatants into mice also increased luminal 5-HT. This increase was due to mast cell secretion of prostaglandin E2 leading to a downregulation of serotonin reuptake transporter on epithelial cells. The 5-HT3 receptor antagonist ondansetron prevented the development of visceral hypersensitivity by IBS fecal supernatants.^7^ Thus, IBS patients have increased neuroactive mediators within the tissue and lumen that increase pain signaling within the GI tract.

Given the heterogenous nature of IBS pathophysiology, drivers of the continued presence of these excitatory mediators may differ in subsets of IBS patients. Since a large proportion of IBS patients report GI symptoms due to food intolerances,^10–13^ specific dietary components may be one of these drivers. Additionally, the gut microbiota may also contribute to the pool of excitatory mediators, as there is growing evidence that gut bacteria modulate visceral sensitivity via signaling to afferent nerves in the GI tract.

## Gut microbiome and visceral sensations

The highly complex and diverse community of microorganisms, collectively called microbiota (or microbiome if we refer to all the microorganisms and their genetic content), comprising archaea, bacteria, viruses, and eukaryotes is mammals’ closest symbiont.^35,36^ The gut microbiota evolves during early life until a unique, subject-specific (fingerprint) adult-like community arises, which is highly resilient and relatively stable throughout life.^37–41^ Inter- and intra-individual variability are very high even among healthy individuals, thus it is difficult to define “the healthy” gut microbiome composition, as this remains a relative state.^42^ Functional redundancy is a key characteristic of the gut microbiota, with different bacterial consortia performing similar functions in different individuals.^43^ However, with the recent advances in technology and analyses software, the importance of strain specificity has emerged as well, meaning that not all strains will have the same impact on health or disease.^42^

The gut microbiota carries out essential functions that the human body is unable to perform,^44,45^ while occupying a unique, nutrient rich niche. The central role of the microbiome is highlighted by studies in germ-free, microbiome depleted, or gnotobiotic animals, which demonstrated that gut microbiota is required for normal gut physiology including gut motility,^46–49^ metabolism, a balanced immune system,^50–56^ and development of the enteric nervous system (ENS).^47,57^ Perception of inflammatory, mechanical and visceral pain is also regulated by the gut microbiota,^5–8,58–60^ however the mechanisms appear to be different depending on the type of pain. While inflammatory and oxaliplatin-induced pain are decreased in absence of bacteria,^58,59^ bacterial pathogens can directly activate sensory neurons and induce mechanical and thermal pain.^60^ Other factors, including sex, may affect the microbial modulation of pain perception as germ-free male but not female mice have increased visceral sensitivity compared to conventionally colonized mice.^5,61^

Modulation of visceral sensitivity by the gut microbiota may occur through the activity of mediators that directly excite nociceptive nerves in the GI tract, such as LPS,^62^ proteases,^34,63^ and histamine,^64^ or through stimulating secretion of excitatory mediators from host cells, such as serotonin from enterochromaffin cells.^65^ Conversely, the microbiota also produces serine proteases that inhibit nociceptive neurons, leading to a decrease in visceral sensitivity.^66^ Thus, the microbiota is capable of both increasing or reducing visceral pain. It is important to keep in mind that the gut microbiota and the host maintain a bidirectional relationship, such that not only does the gut microbiota influence host physiology but the host also influences microbial structure and metabolism. For example, many neuropeptides, such as substance P, calcitonin-related peptide, neuropeptide Y, and vasoactive intestinal polypeptide, have antimicrobial activity.^67^ In addition, nociceptors have been recently shown to both sense commensal and dietary cues.^68^ Moreover, nociceptors can regulate gut microbiota composition, via neuropeptides secretion, which in turn mediate tissue-protective processes in the context of ongoing intestinal inflammation.^68,69^ Thus, the balance of excitatory and inhibitory effects on visceral perception will depend on the specific bacterial strains, the intestinal milieu in which the gut microbiota reside (e.g. active inflammation in the gut, pH, etc.), as well as the interaction between the gut microbiota and luminal factors, including those derived from diet.

## Diet therapy to reduce abdominal pain – clinical perspective

In gastrointestinal disorders with chronic abdominal pain as the predominant symptom, such as IBS, diet has been regarded among first-line treatments as patients complain of adverse reactions to specific foods.^70^ Diet therapies may reduce abdominal pain through multiple mechanisms, including changes in the microbiota. Diet not only impacts microbiome composition, but also influences the microbiome function and metabolite output,^71,72^ which in turn may alter nociceptive signaling within the GI tract. Abdominal pain in some IBS patients may also result from local immune activation triggered by dietary antigens, in both the small^73^ and large intestine.^32^

While diet has become a common therapy to treat patients with chronic abdominal pain, there are currently no biomarkers associating a subset of patients to a response to a specific diet therapy. And although diet is listed among the first-line treatment options for abdominal pain in IBS,^70,74^ the field is fervent with debate on which diet appears to be better.^75–77^ Studying diet as a therapy is challenging as many patients already use diet modification in an attempt to improve their abdominal pain. For example, a recent study found that IBS patients with worse symptoms, primarily driven by increased abdominal pain and symptom-related negative impact on quality of life, are more likely to adhere to exclusion or restrictive diets.^78^ These diets, in turn, result in substantial changes to the gut microbiome composition, which might influence IBS symptoms presentation and perception.^78^ Consequently, this may introduce more heterogeneity into a diet therapy study. Despite these challenges, there has been a significant number of clinical studies investigating diet therapies to treat IBS symptoms. These include traditional dietary advice, a gluten-free diet (GFD), and a diet low in fermentable oligosaccharides, disaccharides, monosaccharides, and polyols (FODMAP), all of which have shown varying degrees of effectiveness for improving symptoms.^76,79–84^ As the GFD and low FODMAP diets are currently the most commonly employed and investigated, we will focus on these two.

### Gluten-free diet (GFD)

Accumulating data suggest that gluten induces symptoms, including abdominal pain, in a subset of IBS patients,^85^ however, the proportion of those reactive to gluten protein may be smaller than originally thought.^86^ Excluding gluten from diet to reduce IBS symptoms has also been questioned due to paucity of randomized, placebo-controlled trials.^87^ However, a recent study suggested that immune reactivity to gluten in the form of anti-gliadin IgG can be used as a biomarker to identify patients with IBS, mainly with diarrhea, who might benefit from a GFD.^80^ The underlying immune mechanisms are unclear, as a recent study using confocal laser endomicroscopy (CLE) concluded that more patients responded to a GFD than to a duodenal application of wheat.^88^ This suggests that some patients, who benefit from a GFD, respond to other components of wheat that do not trigger a response detectable by CLE.^88^ It is also possible that adverse responses detectable by CLE to wheat do not occur in the duodenum, but in the distal small bowel or even colon, thus explaining discrepant results. Alternatively, it has been proposed that fermentable fructans contained in the wheat, rather than gluten, induce symptoms in IBS patients.^86,89^ Reactivity to gluten maybe also be affected by gut microbiota, as several recent studies demonstrated that different bacteria metabolize gluten partially or completely,^90,91^ affecting immunogenicity of gluten-derived peptides.

### Low FODMAP diet

Abdominal pain has been one of the most cited symptoms to improve following reduction in poorly absorbed short-chain carbohydrates, such as FODMAPs.^81–83,92,93^Excessive fermentation of high FODMAP foods leading to gut distension has been proposed as a mechanism contributing to symptom generation in IBS patients. In addition to colonic fermentation, undigested carbohydrates have an osmotic effect increasing water content in the small intestine and colon, that can contribute to gut distension.^94^ However, a study using magnetic resonance imaging (MRI) showed that IBS patients and healthy controls have similar levels of bowel gas and water content, suggesting that abdominal pain is not primarily driven by greater gut distension in IBS patients but rather by visceral hypersensitivity.^95^ Another study demonstrated that increased bowel symptoms after lactulose challenge did not correlate with small intestinal water content or bowel distension, although these differed between IBS patients and healthy controls.^96^ Thus, visceral hypersensitivity is likely a key player, and together with an increase in water content and bowel distension, as well as impaired gas transit and evacuation,^95^ may generate symptoms such as pain and bloating.^94,97^ Although detrimental responses to FODMAPs appear to be driven by peripheral mechanisms, they may also involve central sensitization in response to dietary components as suggested by a recent fMRI study. Following fructan infusion, multiple areas in the brain related to pain, such as the cerebellum, supramarginal gyrus, anterior and midcingulate cortex, insula and thalamus, of IBS patients responded differently compared to healthy controls.^98^

Given that the colonic microbiota ferment FODMAPs, microbial profiles have been implicated in visceral hypersensitivity. A specific microbiota, termed a “pathogenic” microbiota (IBS^P^) due to its pathogenic potential, along with an upregulation in microbial amino acids biosynthesis and carbohydrate metabolism, was suggested to confer the best response to the diet.^99^ On the other hand, a recent meta-analysis concluded that a low FODMAP diet would be recommendable to patients with high colonic methane and SCFA production.^100^ Many other studies have pointed toward gut microbiota composition and function as accountable for the diet response, although no clear unifying pattern has been found (reviewed in details by De Palma and Bercik^84^).

## Diet–microbial interactions underlying visceral hypersensitivity

While the clinical data support a role for diet therapy to reduce abdominal pain, it also suggests that due to the heterogeneity of the patient population, a specific diet may be effective for only a subgroup of patients. Identifying these subgroups has been limited by a lack of mechanistic studies. However, recent studies in animal models have begun to shed light onto possible peripheral mechanisms of how diet–microbiota interactions may underlie the development or perpetuation of symptoms in different subsets of IBS patients.

A higher intake of fermentable carbohydrates has been associated with an increase in Gram-negative bacteria, low-grade inflammation and endotoxemia.^33^ Indeed, a diet low in fermentable carbohydrates reduced fecal LPS in two animal models of stress, as well as improved inflammation, intestinal permeability, and visceral hypersensitivity.^33^ In a follow-up study, the same group showed that a diet high in fermentable carbohydrates in rodents induced TLR4-dependent mast cell activation, which in turn increased colonic permeability.^101^ Importantly, it was demonstrated that IBS-D patients have increased levels of circulating LPS and fecal LPS that is reduced when patients followed a diet low in FODMAPs.^33,101,102^ Intracolonic administration of fecal supernatants from these patients into rats induced visceral hypersensitivity, but this effect was absent when fecal supernatants after a low FODMAP diet were used.^33^ Together, this suggests that in IBS-D patients, foods high in FODMAPs increase luminal LPS due to a higher abundance of Gram-negative bacteria, leading to colonic mast cell hyperplasia and activation, ultimately causing visceral hypersensitivity.

Another putative mechanism underlying visceral hypersensitivity in a subset of IBS patients is related to bacterial histamine production.^64^ Urinary histamine is high in a subset of IBS patients and decreases following a low FODMAP diet.^103^ The source of the increased histamine has not been clearly established as it could originate from dietary sources, be produced by colonic mast cells, or result from metabolic activity of the gut microbiota, as multiple bacteria possess the histidine decarboxylase gene responsible for the conversion of the amino acid L-histidine into histamine. Our recent data demonstrate that the gut microbiota from a subset of patients with IBS can produce large amounts of histamine, that in turn drive an accumulation of mast cells in the colon and visceral hypersensitivity via activation of the histamine 4 receptor.^64^ Moreover, we showed that microbial histamine production is dependent on the colonic fermentative milieu, which determines the pH of the environment. Indeed, mice colonized with a microbiota with high capacity to produce histamine had lower abundance of lactic acid producing bacteria and less lactic acid in the lumen of the colon than mice colonized with a microbiota with lower histamine production. These data suggest that interactions between specific dietary components, such as fermentable carbohydrates, and histamine producing bacteria play a key role in chronic abdominal pain in a subset of IBS patients.

Bacteria may also communicate directly with mast cells, as mice colonized with IBS microbiota demonstrate impairment of the colonic mucus layer, allowing for bacteria to invade colonic tissues, colocalize with mast cells and signal trough both TLR-4 and H4R pathways.^104^ Similarly, *in vitro* experiments showed that mast cells exposed to IBS fecal supernatants had increased release of histamine and prostaglandin E2, an effect that was reduced by exposing mast cells to IBS fecal supernatants obtained after a low FODMPAP diet or when baseline IBS fecal supernatants were exposed to mast cells lacking TLR4.^101^ In parallel, a decrease in serum histamine and tryptase following a low FODMAP diet was observed in a small number of IBS patients.^101^ Collectively, these data suggest that interactions of FODMAPs with the microbiota alter release of a number of neuroactive mediators, of either host and/or microbial origin, which can lead to visceral hypersensitivity.

## Future directions

While diet therapies over the past decade have shown efficacy in improving abdominal pain in subsets of IBS patients, mechanistic data supporting their use has been lacking until recently. Clinical trials of diet therapies present with multiple challenges that can weaken their conclusions,^94^ thus highlighting the need for additional mechanistic insights into which subgroup of patients are most likely to benefit from a diet therapy. Mechanistic studies may also enable identification of key aspects within the diet and/or the microbiota that affect pain signaling. This may help refine a diet therapy that is easier for patients to follow, given that diet therapies to treat abdominal pain are typically diets of exclusion (e.g. low FODMAP diet).

A major limitation in studying diet therapy in IBS patients is the heterogeneity within the patient population. Consequently, studying a subgroup of patients only based on the current clinical classification (e.g. IBS diarrhea predominant or IBS constipation predominant) may underestimate a role for a diet–microbiota interaction driving abdominal pain. Furthermore, a subset of patients may have food-induced abdominal pain due to local immune signaling driven by food antigens, rather than microbiota-related mechanisms.^32^ Therefore, future studies that further phenotype the subgroup of patients to investigate (e.g. presence of a metabolite in the stool, or a well-defined clinical phenotype, such as post-infectious IBS) will lead to better identification of whom is more likely to benefit from a therapy that targets the diet–microbiota interface.

**Figure 1.**
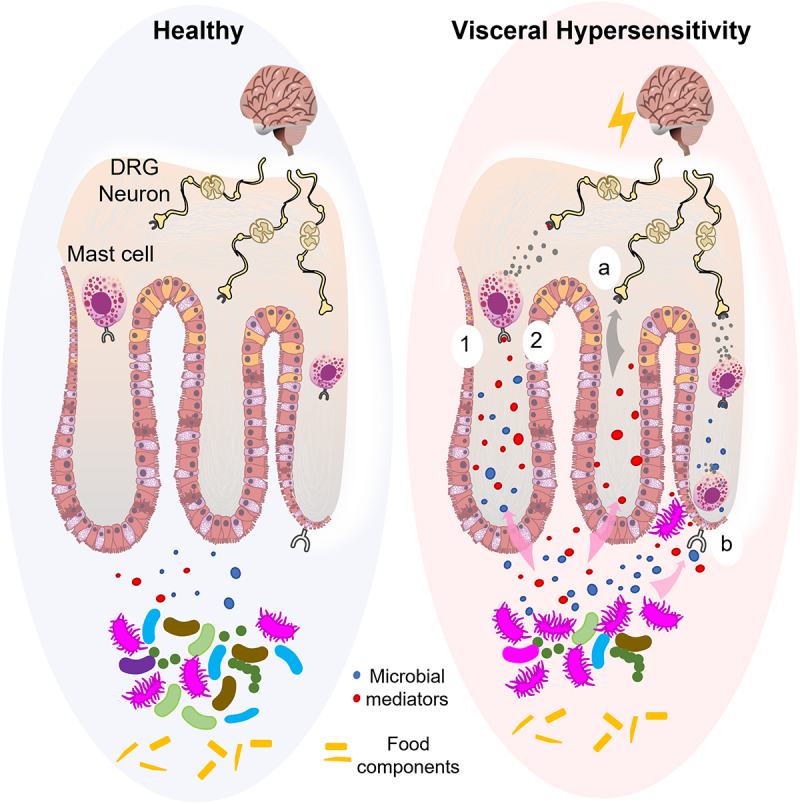
Diet–microbiota interactions leading to visceral hypersensitivity: proposed mechanisms and future directions.

There are a number of approaches that can be taken to study the mechanisms of a diet–microbiota interaction on visceral pain. While clinical trials following patients undergoing a specific diet therapy (or diet challenge to induce symptoms) are necessary, additional translational studies using gut microbiota from the subgroup of interest to colonize germ-free mice to study the effect of a specific diet are ideal to provide mechanistic insight into diet–microbiota interactions in abdominal pain signaling. Alternatively, the microbiota and/or fecal supernatants (which will contain host and microbial mediators), before and after the diet, can be used to study pain signaling in a pre-clinical model. This approach allows a parallel comparison of clinical responses and changes in pain signaling in mice induced by the microbiota and/or fecal supernatants. Furthermore, these approaches may elucidate whether microbial mediators directly activate nociceptive nerves and/or indirectly via activation of host immune cells^23,105^ or epithelial cells^106^ (see Figure 1). In addition to diet therapy, a microbiome-directed therapy aimed at decreasing the abundance of specific bacteria (e.g. high histamine producers) or inhibiting their metabolic activity should also be investigated. Finally, future studies should also elucidate the impact of other known symptom triggers in patients (e.g. stress), when investigating pain signaling resulting from diet–microbiota interactions.

In a healthy host, a rich and diverse gut microbiota interact with food components without subsequent signaling to nociceptive nerves. Recent studies suggest that in patients with visceral hypersensitivity (e.g. IBS), interaction between food components and an imbalanced gut microbiota may increase nociceptive nerve activity via: (1) secretion of immunomodulators (e.g. histamine) or (2) increased abundance of LPS, that in turn elicit a host immune response, such as secretion of mast cell mediators (i.e. histamine, tryptase and prostaglandins) leading to activation of nociceptive nerves. Other potential mechanisms that require further investigation include (a) direct activation of nociceptive nerves by microbial mediators and (b) activation of epithelial cells by microbial mediators leading to secretion of factors (e.g. proteases) that ultimately drive nociceptive nerve activation.

## Conclusions

Diet therapies may offer relief of chronic abdominal pain to some patients, such as those with IBS. However, the restrictive nature of many diet therapies might not be sustainable in the long-term due to the diet complexity, as well as its potentially negative long-term effects on the microbiome. Only recently have studies begun to elucidate mechanisms of how diet-microbiota the mechanisms of how diet–microbiota interactions may induce visceral hyperalgesia. Therefore, further studies are needed to identify the patients with chronic abdominal pain who would benefit most from a diet directed and/or a microbiome-directed therapy.
